# Sequelae of Infective Endocarditis: Ruptured Aortic Root Abscess in a 38-Year-Old Female With Complicated Infective Endocarditis

**DOI:** 10.7759/cureus.23147

**Published:** 2022-03-14

**Authors:** Imran A Qureshi, Sarah Ashraf, Mohammad Pervez, Saulat Fatimi

**Affiliations:** 1 Cardiothoracic Surgery, Aga Khan University, Karachi, PAK; 2 Surgery, University of Toronto, Toronto, CAN

**Keywords:** aortic valve replacement, valve abscess, acinetobacter, ruptured aortic root abscess, infective endocarditis

## Abstract

A 38-year-old female with no known comorbidities or previous history of heart disease presented to the hospital with a three-day history of drowsiness and shortness of breath. Transthoracic echocardiography was performed, which showed large vegetations on aortic and tricuspid valves. In addition, there was severe aortic regurgitation with a possible abscess on the non-coronary cusp of the aortic valve.

The patient was admitted, and a provisional diagnosis of disseminated tuberculosis, Infective endocarditis (IE), and sepsis was made. Surgical intervention was planned. Intraoperative findings revealed that a fistula had formed connecting the aorta and right atrium, which was closed with an autologous graft derived from the patient’s pericardial tissue. Vegetations were removed, and the aortic valve was replaced with a metallic valve.

This case report presents a patient with complicated IE with a ruptured aortic root abscess. Mechanical complications associated with IE, such as in our case, are rare among patients with IE. However, surgical intervention should be considered as an option in complicated cases of IE when standard therapy fails.

## Introduction

Infective endocarditis (IE) can be acute or subacute. It can result from a bacterial, fungal, or viral infection of the endocardial surface of the heart. Rheumatic heart disease is a major risk factor for developing IE in low- and middle-income countries (LMICs). Other risk factors include congenital heart disease, valvular disease, diabetes, cancer, and IV drug abuse [1]. In Pakistan, rheumatic heart disease is particularly prevalent in children aged 5-16 years old, especially in rural areas [2]. This coincides with the younger age of presentation of IE in our population [3,4].

IE has a high prevalence in developing countries worldwide, with 2.2 per 1000 cases in south-central Asia. An interesting feature of IE cases from developing countries is the high prevalence of culture-negative endocarditis, which was the case with our patient. This further complicates the diagnosis and management of such patients [5].

Patients with IE often present with non-specific symptoms such as fever with chills, anorexia, weight loss, malaise, headache, nausea, myalgia, shortness of breath, cough, or joint pains. The most common causative organism causing IE is Staphylococcus aureus [6]. IE is diagnosed using the modified Duke clinical diagnostic criteria.

The following case is of a patient with IE that presented with shortness of breath and drowsiness, which progressed to sepsis. This case report intends to shed light on the mechanical complications associated with IE and the role of surgical intervention in improving clinical outcomes in patients with complicated IE. Informed consent was taken from an authorized relative on the patient’s behalf to publish their case.

## Case presentation

A 38-year-old female with no known comorbidities or previous history of heart disease presented with a three-month history of low-grade fever, generalized body weakness, decreased appetite, joint pain, and weight loss. In addition, she was previously diagnosed with severe anemia with a hemoglobin of 4.8 g/dL and had received a blood transfusion. Over the span of three months, her condition gradually worsened, and she developed shortness of breath and became drowsy, for which she was admitted to the hospital. A pan systolic murmur was heard at the apex radiating to the axilla on initial examination. A provisional diagnosis of IE and sepsis was made. Differential diagnoses included disseminated tuberculosis, autoimmune serositis, and myeloproliferative disease. 

**Figure 1:**
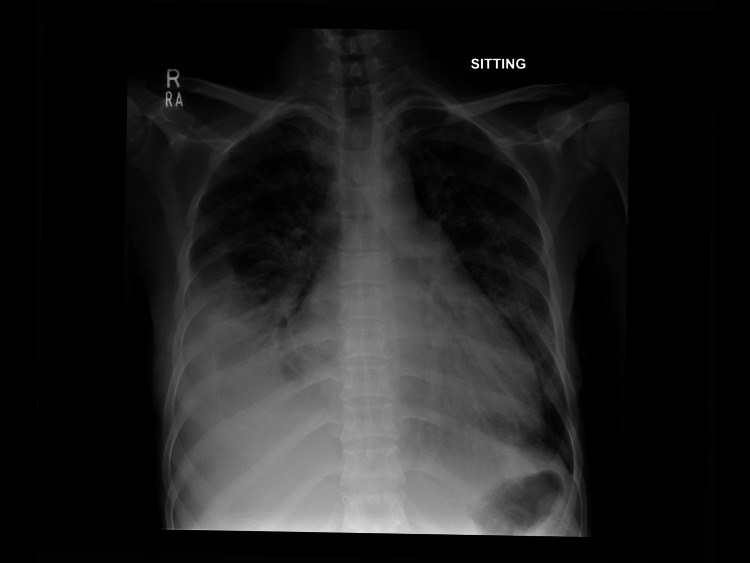
Chest X-ray in AP position showing a magnified cardiac silhouette and moderate right-sided pleural effusion noted with possible underlying atelectasis. There is perihilar congestion with a prominence of upper lobe vessels noted.

**Figure 2:**
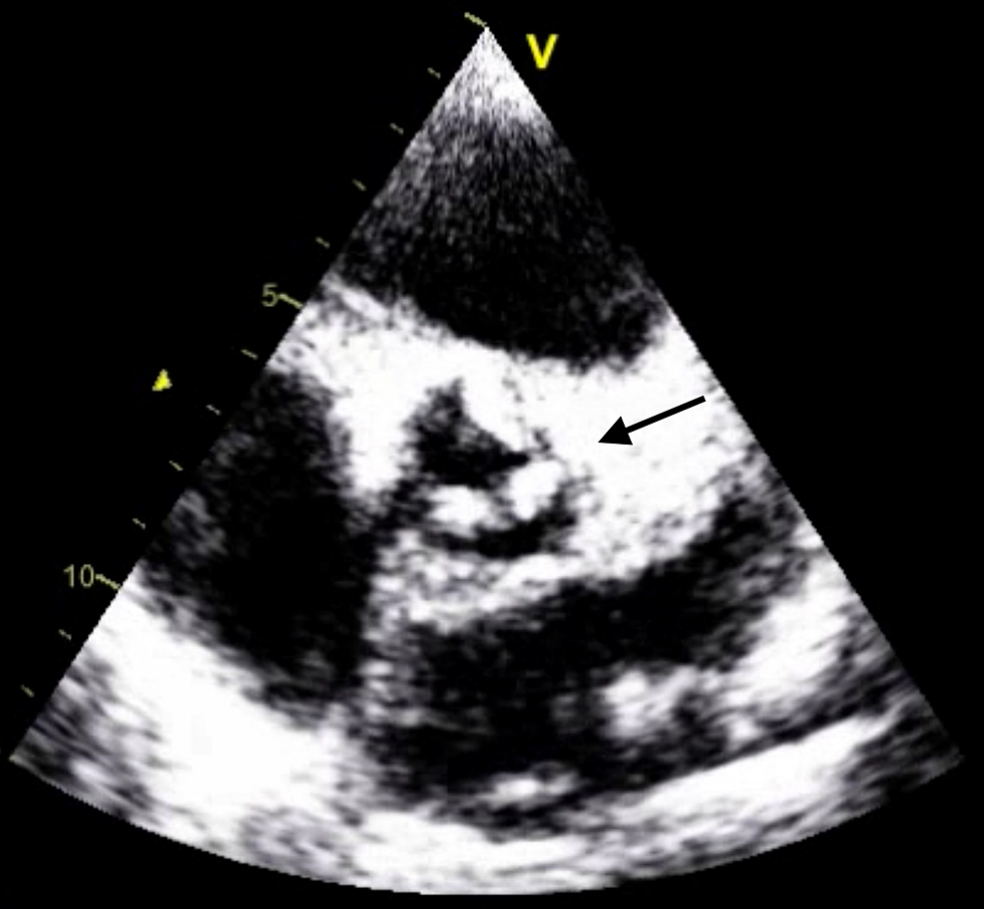
Large echogenic densities were noted on the aortic valve on the transthoracic echocardiogram.

Chest X-ray (Figure 1) revealed a magnified cardiac silhouette along with perihilar congestion with a prominence of upper lobe vessels noted and a right-sided pleural effusion with possible underlying atelectasis. In addition, there was hepatomegaly with a dilated portal and hepatic veins suggestive of congestive hepatopathy with multiple small low-attenuation lesions in the spleen. Her pro-brain-type natriuretic peptide (BNP) level was found to be 38,132 pg/mL. The presence of hepatitis B and C, systemic lupus erythematous (SLE), Brucella, and Celiac disease was ruled out. Blood cultures were negative for the growth of any microorganisms. Transthoracic echocardiography (Figure 2) showed mild-to-moderate mitral regurgitation, moderate aortic regurgitation, and moderate-to-severe tricuspid regurgitation with severe pulmonary hypertension. Large echogenic densities were noted on the aortic valve, and mobile echogenicities were present on the tricuspid valve. The abnormal flow was noted between the aorta and right ventricle, which was either suggestive of a ventricular septal defect (VSD) or a fistula. Diagnostic aspiration and cytology of ascitic fluid revealed increased polymorphs suggestive of acute inflammation with no malignancy. The ascitic fluid culture was negative for acid-fast bacilli and other microorganisms.

**Figure 3:**
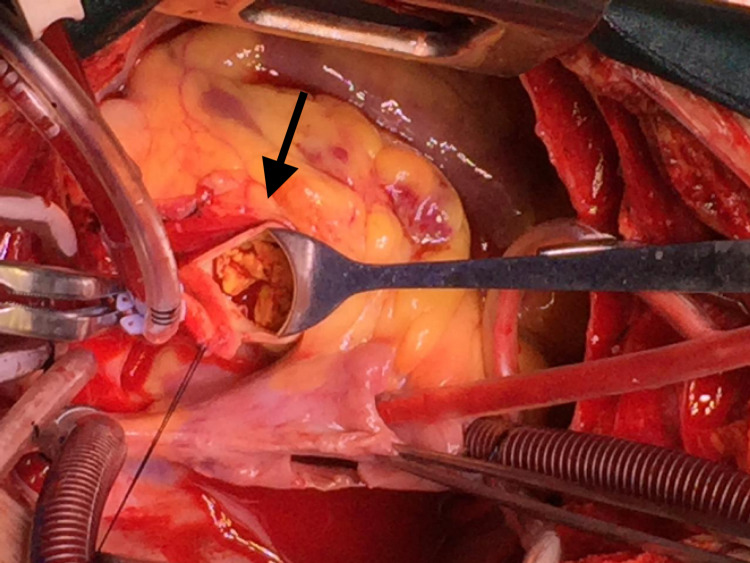
Endocarditis caused an abscess approximately a millimeter below the annulus of the non-coronary cusp, which opened into the right atrium.

**Figure 4:**
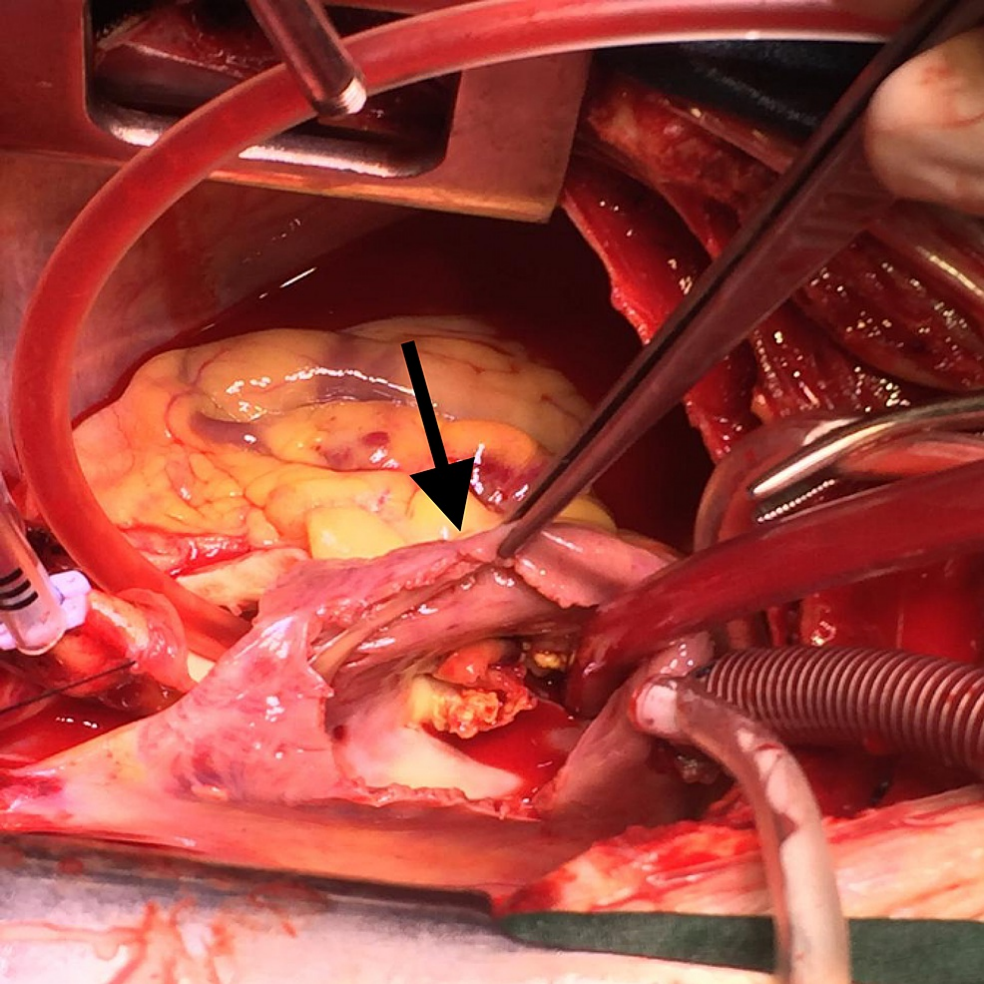
The aortic valve totally destroyed with vegetations, causing aortic stenosis and regurgitation.

On the fifth day of hospitalization, she developed metabolic acidosis and septic shock. Subsequently, she was intubated and shifted to the medical ICU, where the patient developed acute kidney failure. Transesophageal echocardiography (TEE) revealed large vegetations on the aortic valve with a possible abscess on non-coronary cusp and severe aortic regurgitation. In addition to this, there were vegetations on the tricuspid valve with moderate tricuspid regurgitation and severe pulmonary hypertension. Due to the patient’s progressively worsening clinical condition, surgical intervention was planned on the 10th day of hospitalization. The surgery involved the replacement of the aortic valve with a 19 mm mechanical valve and debridement of vegetations on the tricuspid and aortic valves. Intraoperatively, an abscess was noted approximately a millimeter below the annulus of the non-coronary cusp, which opened into the right atrium (Figure 3). Another finding was that the aortic valve was totally destroyed with vegetations, causing aortic stenosis and regurgitation (Figure 4). The abscess was drained and washed out with normal saline, and the defect was covered with a pericardial patch. Tricuspid valve vegetectomy was done with the leaflets partially left intact. Postoperatively, the patient was shifted to the cardiac ICU on inotropic support. Postoperative transthoracic echocardiography demonstrated a severely dilated left atrium and moderate mitral and tricuspid regurgitation. A prosthetic valve was also noted in the aortic position with significantly increased gradients. She was successfully extubated on her first postoperative day. Gram-positive bacilli were identified in the smear of the removed aortic valve that failed to grow on culture. Blood cultures taken on the 10th postoperative day showed growth of pan-resistant Acinetobacter, sensitive only to colistin. On the third postoperative day, the patient had to be intubated again due to carbon dioxide retention and worsening tachypnea. The patient developed congestive hepatopathy, and her liver function was deranged. This was followed by pancytopenia and progressively worsening renal failure. Subsequently, the patient developed septic shock, which eventually led to her demise. 

## Discussion

IE is an infection of either the endocardial surface, native or prosthetic cardiac valve, or any indwelling cardiac device. Patients with IE often present with non-specific symptoms such as fever, malaise, weight loss, backache, and dyspnea with focal neurological deficits. Despite advances in management and treatment, mortality associated with IE remains high [7]. The most common pathogens involved in the pathogenesis of IE include Staphylococcus aureus followed by Streptococcus pneumonia, enterococci, and HACEK organisms (Haemophilus, Aggregatibacter, Cardiobacterium hominis, Eikenella corrodens, Kingella species) [8].

Acinetobacter species are most commonly associated with pneumonia, surgical site infections, and catheter-related bloodstream infections [9]. Treatment of infections caused by Acinetobacter continues to be a challenge due to its resistance patterns [10]. Infections due to Acinetobacter have shown higher mortality rates than infections caused by other nosocomial pathogens [11]. The prognosis is further worsened by an inappropriate choice of empirical antibiotics [12]. In a study done in a tertiary care hospital in Pakistan, Begum S et al. found Acinetobacter baumannii to be the leading cause of nosocomial infections and reported a high prevalence of multidrug resistance amongst A. baumannii isolates [13].

Management of IE involves long-term administration of bactericidal antibiotics with an appropriate regimen based on the type of organism isolated. Indications for surgical valve replacement or repair is done in the presence of acute complications or complications not managed with antibiotic therapy alone. These include abscess, recurrent embolic events, presence of a multidrug-resistant organism, or persistent bacteremia [14]. Surgical management of IE is highly variable and can involve surgical removal of the valve with valve repair to a more complex procedure involving radical resection and replacement of the valve with perivalvular tissue. However, even after appropriate surgical intervention, the rate of developing complications with early mortality still remains high [15].

## Conclusions

The diagnosis and management of IE still remain a challenge due to the highly variable presentation of the disease. The choice of empirical antibiotics and the decision to resort to surgical intervention in a patient with multiple complications of IE involves considering factors such as patient characteristics, presence of comorbidities, and disseminated disease. Despite this, the prognosis of patients who have developed mechanical complications secondary to IE remains poor, as highlighted in this case. Early surgery for native valve endocarditis may be associated with lower long-term mortality. As far as the optimal timing of when the surgical intervention should be planned still remains undecided.
